# Can an Integrated Science Approach to Precision Medicine Research Improve Lithium Treatment in Bipolar Disorders?

**DOI:** 10.3389/fpsyt.2018.00360

**Published:** 2018-08-21

**Authors:** Jan Scott, Bruno Etain, Frank Bellivier

**Affiliations:** ^1^Institute of Neuroscience, Newcastle University, Newcastle upon Tyne, United Kingdom; ^2^Centre for Affective Disorders, Institute of Psychiatry, Psychology and Neuroscience, King's College London, London, United Kingdom; ^3^Faculté de Médecine, Université Paris Diderot, Paris, France; ^4^AP-HP, Groupe Hospitalier Saint-Louis-Lariboisière-Fernand Widal, Paris, France; ^5^INSERM, Unité UMR-S 1144, Variabilité de Réponse aux Psychotropes, Université Paris Descartes-Paris Diderot, Paris, France; ^6^AP-HP, Groupe Henri Mondor-Albert Chenevier, Pôle de Psychiatrie, Créteil, France; ^7^INSERM, Unité 955, IMRB, Equipe de Psychiatrie Translationnelle, Créteil, France

**Keywords:** bipolar disorders, lithium, mood stabilizers, prediction, response, phenotype, biomarkers, personalized

## Abstract

Clinical practice guidelines identify lithium as a first line treatment for mood stabilization and reduction of suicidality in bipolar disorders (BD); however, most individuals show sub-optimal response. Identifying biomarkers for lithium response could enable personalization of treatment and refine criteria for stratification of BD cases into treatment-relevant subgroups. Existing systematic reviews identify potential biomarkers of lithium response, but none directly address the conceptual issues that need to be addressed to enhance translation of research into precision prescribing of lithium. For example, although clinical syndrome subtyping of BD has not led to customized individual treatments, we emphasize the importance of assessing clinical response phenotypes in biomarker research. Also, we highlight the need to give greater consideration to the quality of prospective longitudinal monitoring of illness activity and the differentiation of non-response from partial or non-adherence with medication. It is unlikely that there is a single biomarker for lithium response or tolerability, so this review argues that more research should be directed toward the exploration of biosignatures. Importantly, we emphasize that an integrative science approach may improve the likelihood of discovering the optimal combination of clinical factors and multimodal biomarkers (e.g., blood omics, neuroimaging, and actigraphy derived-markers). This strategy could uncover a valid lithium response phenotype and facilitate development of a composite prediction algorithm. Lastly, this narrative review discusses how these strategies could improve eligibility criteria for lithium treatment in BD, and highlights barriers to translation to clinical practice including the often-overlooked issue of the cost-effectiveness of introducing biomarker tests in psychiatry.

## Introduction

Although Hippocrates (fourth century BC) is often described as the “Father of Modern Medicine,” the reality is that the science of disease prevention and treatment is a relatively recent phenomenon. Until the twentieth century, careful observation and clinical judgement underpinned most medical practice. In the twenty first century, clinical practice guidelines (CPGs) began to appear with increasing frequency. These CPGs were based on the principles of evidence-based medicine (EBM) and emphasized that treatment recommendations for physical and mental disorders should be founded on systematic assessment of the strength of evidence available (which was graded hierarchically e.g., meta-analyses were ranked above individual studies, etc.) (1, 2). Whilst CPGs were an improvement on subjective opinions, treatments were invariably recommended based on the “average response” amongst individuals recruited to randomized controlled trials (RCTs) and it became increasingly clear that this “one size fits all” approach did not meet the needs of some of the most disabled patients (1, 2). Recently, attention has shifted toward the potential utility of personalized or precision medicine in clinical practice (3, 4). There are subtle differences in the meaning of these terms (see Table 1), but both personalized and precision medicine share the same goal, namely to tailor diagnostic and treatment decisions to each patient by utilizing information about individual phenotypes and genotypes (3, 4).

**Table 1:** Definitions of key concepts.

**Concept**	**Definitions and Observations**
Evidence-Based Medicine (EBM)	EBM is an approach to medical practice intended to optimize decision-making by integrating the best research evidence with clinical expertise and patient values (1, 2). Sackett et al. (1) state that evidence is viewed epistemologically e.g., only strong evidence (from meta-analyses, systematic reviews, and randomized controled trials) can yield strong recommendations. However, Perna et al. (7) note that EBM emphasizes the use of scientific evidence regarding the most effective intervention for the “average” patient with a specific diagnosis, leading to criticism of the utility of EBM in day-to-day practice (2).
Personalized Medicine	Personalized Medicine proposes to establish clinical decisions based upon a patient's individual profile, tailoring the treatment to their characteristics and needs (4, 7). It is argued that the term “personalized” should be replaced by “precision” as the former term may also be used e.g., in psychotherapy to describe the formulation of an individual's problems or as a generic term referring to clinical management (i.e., it does not specify the links to pathophysiological mechanisms).
Precision Medicine	The Precision Medicine Initiative defined precision medicine as “an emerging approach for disease treatment and prevention that considers individual variability in genes, environment, and lifestyle for each person” (4).
Stratified Medicine	Stratified medicine focuses on the identification of biomarkers or psychological tests to stratify patients in smaller treatment relevant subgroups. Wium-Andersen et al. (3) note that, unlike precision medicine, stratification does not require complete understanding of the etiology of the underlying illness and that it may coexist alongside conventional diagnostic systems, where patients are first diagnosed and later stratified into putative treatment subgroups.

Precision medicine has yielded important breakthroughs in several branches of medicine, such as the development of drugs that target cells containing large amounts of HER2 (human epidermal growth factor receptor 2) in breast cancer (5). However, there are many clinical, methodological, and regulatory challenges to the translation of data about individualized profiles and diagnostics into personalized interventions (4, 5). Recognition of these challenges and concerns about delays in the introduction of more effective, tailored interventions for patients led the European Union (EU) to propose a new funding stream for the development of personalized approaches to complex diseases with high prevalence and high economic impact (6, 7). Most major mental disorders match this description, and there is an acknowledged need to provide reliable assistance to clinicians to help them to customize treatment decisions in psychiatry (7, 8). For example, psychiatrists, patients, their families and significant others would welcome a more nuanced approach to the use of mood stabilizers in individuals with bipolar disorders (BD), especially greater precision in prescribing of long-term treatment with lithium in bipolar-I-disorder (BD-I).

There are several systematic reviews of potential biomarkers of response to lithium and emerging ideas on research strategies that may aid identification of biomarkers or treatment selection. However, we did not identify any reviews that specifically focused on the broader conceptual and strategic issues (e.g., how to integrate high quality biomarker research with more reliable and valid clinical observations; whether to search for single biomarkers or multi-modal sets of markers). In essence, this paper does not review new findings, but rather reconsiders the state of the art and what can be learnt and applied to future research in the field of biomarker research. Given this apparent gap in the literature, we undertook a narrative review to consider these broader, but potentially critical issues and how they may inform efforts to translate research findings in precision prescribing.

This review begins by briefly highlighting the arguments for, but difficulties in, applying precision medicine first to the diagnosis of BD subtypes and second to the prescribing of mood stabilizers. Next, we consider core components and underlying principles that could inform viable research into precision psychiatry, using the example of the prescription of lithium in BD-I. Several publications have highlighted details of and evidence for putative biomarkers [e.g., (9–12)], so rather than replicate those reviews, we primarily focus on broader, strategic issues regarding the application of precision medicine research to psychiatry. We describe these in terms of “bottom-up” approaches (namely, the under-rated role of systematic assessment of baseline characteristics, clinical phenotypes and longitudinal monitoring of illness activity and treatment response, etc.) and the importance of combining these with “top-down” approaches (highlighting some of the guiding principles for examining biomarkers). We conclude by identifying some of the key considerations in implementing “precision” that may be relevant to BD research, to psychiatry and potentially to general medicine as well.

## Materials and methods

Literature searches were undertaken to identify publications (reviews, discussion papers, individual studies, policy documents, conference proceedings, etc.) addressing the topics of BD (diagnosis, prediction of treatment response, and outcome), mood stabilizers (prescribing, efficacy, and prediction of response to lithium), and precision, personalized, or stratified medicine (as defined in Table 1). Relevant information was extracted, and key concepts were synthesized under the headings reported in this narrative review. However, we explicitly focused on broader conceptual and methodological issues and aimed to avoid reporting information or evidence that had been reviewed in other publications. Additionally, the authors incorporated some of the insights gained from the development of a multicentre grant application entitled R-LiNK (Response to Lithium Network); that has received EU funding (http://www.r-link.eu.com). Lastly, the extant literature was examined to identify some key lessons that have been learnt or may need to be learned to enable precision in psychiatry and in the treatment of BD.

## Results

We begin by commenting on the diagnosis of BD and the more narrowly defined subtype of BD-I, then consider definitions of and prescribing of mood stabilizers, before focusing on precision medicine approaches to the prediction of response to lithium in BD-I.

### Bipolar disorders

The term BD refers to a group of disorders that are primarily characterized by changes in mood, activity and energy (13). The two main subtypes are BD-I (characterized by at least one episode of mania) and BD-II (characterized by at least one episode of major depression and of hypomania), but other variants, referred to as the BD spectrum, are recognized. The morbidity associated with BD can partly be explained by the early peak age of onset (of 15–25 years), the prevalence (1–4% of the global population depending on range of BD spectrum included) and the high rates of physical and mental comorbidities; also, BD is a leading cause of suicide (14, 15). Overall, BD is ranked 6th in the global burden of diseases in working age adults (14) and 4th amongst youth aged less than 25 years (15).

As well as the clinical and social impairments experienced by individuals with BD and the stress and distress experienced by their families and significant others, a recent study from the USA estimated the total economic cost of BD-I was greater than $200 billion (16). The largest contributors to excess costs were caregiving, direct health care and unemployment; findings that highlight the importance of early diagnosis and the need to optimize therapeutic strategies.

Early diagnosis of BD is hampered by the lack of objective laboratory tests in psychiatry. As such, accurate diagnosis of any mental disorder is dependent on careful observation of presenting signs and symptoms, skillful history-taking, and clinical expertise. Unfortunately, even when international criteria are employed, the reliability of diagnoses varies significantly (17). Diagnostic precision is diminished because the symptoms and signs of one mental disorder may overlap considerably with several other diagnostic categories (18). In BD, this problem is compounded by the fact that symptoms vary greatly between the different syndromes included within the BD spectrum. Although there are shared clinical features across the BD spectrum, current research indicates that these disorders probably represent heterogeneous syndromes resulting from multiple etiological processes, rather than comprising a specific disease category (19, 20).

Given the lack of diagnostic reliability and limited biological validity of the broad category of BD and its spectrum, further important research continues as in the long-term precision approaches in psychiatry will aim to commence with precision diagnostics. However, the majority of current studies of the application of precision medicine in BD should be limited to a more narrowly defined subgroup. The obvious subtype to target is BD-I as this is one of the three most reliable diagnoses in psychiatry (17) and objective measurements are available of current mental state, such as activation and sleep-wake cycle (21). However, it must be emphasized that this relatively more clinically homogeneous BD-I diagnostic subtype lacks a disease signature and current animal models demonstrate low predictive power (5); so personalized diagnostics are not viable and will remain an aspiration for the foreseeable future for BD. As such, we suggest that efforts might best be directed toward the identification of “treatment-relevant” subgroups (also referred to as stratified medicine; see Table 1) for prescribing recommended medications for BD-I, such as mood stabilizers.

### Mood stabilizers

It is generally accepted that the term mood stabilizer refers to a category of medication that shows at least two of the three following properties: anti-manic, antidepressant and prophylactic, without increasing the risk of episodes of the opposite polarity (22). International CPGs repeatedly identify lithium as a “gold standard” mood stabilizer treatment, with robust evidence for its efficacy in preventing BD relapses and rehospitalizations and reducing suicidality (23–25). Lithium is also prescribed as an acute treatment for mania and as an adjunct to traditional antidepressants in acute major depressive episodes. Another potential advantage is that a 1-month supply of lithium only costs about one dollar compared with $15 per month for olanzapine and $60 per month for valproic acid (26).

Despite being regarded as a first line, cost-effective treatment, there is a significant gap between the research efficacy and clinical effectiveness of lithium. Only about 30% of lithium-treated patients show an excellent long-term response and the short-term benefits of acute lithium treatment do not reliably predict the outcome of prophylaxis. Overall, it can take 18–24 months to determine that there has been a significant reduction in the frequency or severity of BD episodes or a clinically meaningful decrease in illness activity. The extended time frame for ascertaining a true positive “good response,” the prevalence of sub-optimal outcomes and the narrow therapeutic window for lithium, plus its perception as less safe than newer compounds, have all probably contributed to the reported decline in lithium use (26–28).

Robust biological predictors of continuation and maintenance treatment response (or of tolerability) remain elusive (26). Further, the clinical predictors identified by psychiatrists (e.g., family history of lithium response, BD subtype, etc.) are not consistently supported by the empirical literature (26, 29–31) and cannot be employed as reliable eligibility criteria for lithium prescribing. Critics of EBM and CPGs argue that they do not assist in the identification of moderators and mediators of lithium response or of non-response because they focus on highly selected samples of patients recruited to RCTs. Overall, findings from these current approaches lack external validity or generalizability to real world clinical settings (32).

Presently, many clinicians and patients are dependent on a “trial and error” approach to prescribing lithium and determining its effectiveness. This strategy is problematic as the prolonged trial combined with concerns about side-effects may increase the risk of lithium non-adherence, which compounds the likelihood of treatment failure (33, 34). Understandably, there is an increased impetus toward research that enables targeting of lithium treatment toward subgroups of patients who are most likely to benefit. Given the complexity of BD-I and the limited understanding of the mechanisms of action of lithium (28), the search for these “responder/non-responder” subgroups or phenotypes needs to combine the systematic exploration of socio-demographics, clinical characteristics, and course of illness, with genetics, omics, neuro-imaging, and other biomarker tests, etc. This might allow clinicians and patients to predict the likelihood of response (or of non-response or intolerance) to lithium prior to the initiation of mood stabilizer treatment or within the first few months of its commencement.

### An integrated science approach to response prediction

As precision psychiatry research is in its infancy, we describe some of the options for studying precision or stratified medicine approaches to the prediction of response to lithium in BD-I. We suggest that it is more realistic for research programs to integrate clinical assessment and monitoring (so-called “bottom up”) approaches with applied biological research and analytic approaches (so-called “top down”) (35, 36). Each strategy is insufficient for identifying putative predictors of response alone, but their joint application may lead to progress. We briefly highlight some key considerations, beginning with clinical strategies.

#### Bottom-up approaches

Three relevant clinical considerations are (i) sample selection; (ii) assessment of past history and current diagnosis, and prospective longitudinal monitoring of symptoms and illness dimensions (including patient related outcomes); (iii) assessment of lithium response and strategies for minimizing confounding of treatment non-response with non-adherence.

##### Sampling

A recognized problem of studies of predictors and biomarkers for treatment response is that they are largely based on secondary analyses of data from samples included in efficacy RCTs (37). This, plus other limitations in the recruitment process (such as convenience sampling and small sample sizes), has produced a number of biases in reported findings (38). Whilst data from efficacy studies will show a better signal-to-noise ratio, there is an argument that predictors of treatment response in psychiatry might best be identified from prospective cohort studies of large clinically representative samples that are purposefully designed. This approach is being used in R-LiNK which is a project that is being undertaken in 15 clinical centers across eight EU countries that aims to study individuals with BD-I who clinicians have identified as candidates for a trial of lithium (according to the recommendations in the CPG employed at those centers). Whilst the pragmatic design has some drawbacks, the prospective assessment of treatment response in about 300 patients over 2 years will offer important insights into the real-world effectiveness of lithium, which increases the translational potential of any findings regarding early predictors of response.

##### Clinical assessments

Although clinical syndrome subtypes are insufficient for personalizing psychiatric treatment (3), they are still relevant for developing a detailed clinical picture of each persons' experience of the disorder being studied. This includes structured clinical interviews that allow longitudinal assessment of lifetime and current diagnoses as well as allowing researchers to reconstruct the illness trajectory, prior history, etc. In BD-I, this includes determination of precursors and patterns of illness episodes (e.g., antecedents and symptom profiles of familial and non-familial BD), polarity of BD onset, predominant polarity, history of psychotic symptoms, frequency, and severity of episodes, inter-episode symptoms, and functioning, etc.

The choice of diagnostic instrument is not usually contentious (e.g., existing structured clinical interviews offer a detailed assessment of BD as well as other psychiatric comorbidities), and these interview schedules can be combined with tools that screen for medical comorbidities, etc. However, a weakness of previous studies of psychotropic response biomarkers is that they have paid insufficient attention to the rating scales used to assess symptoms and how these change during treatment. Any change in illness activity post-initiation of lithium needs to be captured by measures that enable observers to determine the evolution of symptoms and syndromes and ensure a detailed picture of BD-I before and after the introduction of lithium can be constructed. This is important as precision psychiatry research should aim to be compatible with other approaches to identifying treatment response phenotypes, such as the R-DOC framework (39) which highlights the importance of examining trans-diagnostic illness dimensions that are grounded in neuroscience, e.g., cognitive, arousal, and regulatory systems, etc.

Regarding BD-I studies, the rating scales selected for monitoring disease progression during exposure to lithium treatment need to clearly reflect the core dimensions of the current diagnostic criteria (mood, activity, energy) and give adequate weighting to the symptoms that are especially likely to change during lithium treatment, as these may represent clinical markers of response. It should be borne in mind that most mania and depression rating scales were developed about 30–40 years ago and demonstrate considerable heterogeneity in the range of symptoms assessed and in the underlying assumptions about the nature of BD episodes (21, 38). For example, the Young Mania Rating Scale (YMRS) was introduced at a time when elation or irritability were the criterion A symptoms for BD (not mood and activation as described currently) (13) and, unsurprisingly, YMRS ratings correlate poorly with objective measures of activation (21, 40–42). Likewise, the 6-item version of the Hamilton Rating Scale for Depression (HRSD-6) and the 16-item version of Inventory of Depressive Symptoms give equal weighting to each core dimension of depression and have better psychometric, IRT (item response theory) and clinimetric profiles than other scales used to assess depression in BD (40, 43, 44). These may seem like lower order issues, but they take on greater significance when considered in the context of the fact that change in activation or sleep-wake cycle may occur early in lithium responders or may be early warning signs of relapse when lithium is withdrawn (42). Thus, selection of rating scales to monitor illness activity longitudinally is a critical but often overlooked element of biomarker research.

Whilst repeated observer assessments of course of illness and treatment response are important, consideration should be given to how these measures can be supplemented by subjective ratings and patient related outcomes (PRO). Techniques such as PRO can ensure that additional measures of functioning or outcomes that are important to individual patients (such as “personal recovery”) are also included (45). Whilst this may extend the range of response categories that are considered for analysis, it improves the likelihood that a study will be valued by patients as well as clinicians and researchers. Symptoms or items that are identified for subjective rating can be included in ecological momentary assessments (EMA) when feasible, to allow more detailed analysis of response patterns and variability in treatment outcomes (21, 46). In BD-I, these measures may be combined with objective real-time monitoring of sleep-wake cycle and activity patterns using actigraphy (21).

Lastly, clinical assessments may extend beyond symptom ratings to include other measurements such as neuropsychological profiles. The latter has been linked to biological measures of response [e.g., structural and functional magnetic resonance imaging (MRI)] and may show direct or indirect (via medication non-adherence) associations with treatment response (47).

##### Treatment response and adherence

A critical element of any study of treatment response is to carefully operationalize definitions of this concept. As lithium is a prophylactic treatment, then reduction in BD relapses during a demarcated follow-up period is typically the most important parameter. However, some studies categorize lithium response based only on retrospective assessment, and even prospective studies may fail to employ (or describe) any consensus definitions of lithium response.

As a first step, researchers need to decide if response is synonymous with e.g., absence of any illness episodes meeting syndromal criteria over a given timeframe; achieving symptom remission for a specified period; time to remission (or to relapse) after commencing lithium; time to “treatment failure” (e.g., stopping lithium; introduction of another mood stabilizer; etc.). Further, each of these conceptualizations of response may need to be considered in the context of vulnerability to adverse effects or side effects. Thus, researchers need to provide a detailed description of the criteria employed to define response so that others can consider the reliability and validity of the definitions (against which the biomarkers are bench-marked), the applicability to their own clinical or research setting and to ensure that findings from other studies can be compared and contrasted accordingly.

Another overlooked element of the definition of treatment non-response is that it is not always sufficiently differentiated from non-adherence. Whilst these concepts overlap, it is worth noting that if a patient is non-adherent with lithium but appears to have a good outcome during prospective monitoring, then any identified predictors may be markers of illness trajectory (i.e., the predictor variables may be identifying individuals with a good prognosis independent of the treatment being assessed). Further, existing evidence, e.g., from genetics studies, suggests that the way in which these two variables (response and adherence) are considered may influence reported findings and which markers may be regarded as significant (48, 49). Interestingly, the TRIPP (treatment response and resistance in the psychosis) working group advocate obtaining measures of medication adherence and employing minimum adherence criteria before classifying patients as non-responders (50). In R-LiNK, measures of plasma levels of lithium and observer and self-reported assessments of medication adherence will be employed. These will be used alongside regular measurement of health beliefs that have previously been shown to identify individuals at high risk of becoming partially or non-adherent in the near future (51). In this way, it is possible to minimize confounding of non-response and non-adherence (50) and to consider the introduction of simple evidence-based clinical strategies to optimize medication adherence (52).

#### Top down approaches

There has been a rapid expansion in research into precision psychiatry, especially regarding biomarkers (measurable characteristics that reflect biologic function or dysfunction, response to a therapeutic measure, or indication of the natural progression of disease) (12). A scoping exercise of biological predictors of lithium response indicates that researchers usually explore one or more of three types of biomarkers (53):

- functional markers, which can be measured e.g., by neurocognitive tests or neuro-imaging of neural networks;
- structural markers that assess the topography of brain architecture;
- metabolic/molecular markers, such as omics and including microRNAs that reflect glial and neuronal dysfunction.

To date, many lithium biomarker studies are domain specific (e.g., focused only on neuroimaging or omics, etc.). Although this research contributes to the overall understanding of the state of the art, there are potential problems in interpretability of multiple individual studies (38). We concur with Trivedi et al. (54), who suggest that it is unlikely that there is a single biomarker for psychotropic response or non-response that is based only on a single dimension or modality (e.g., genetics, omics, neuroimaging, neuropsychology, or clinical presentation). As such, the rest of this section focuses on three important considerations for research on precision prescribing: multidimensional phenotyping; analytic strategies for response prediction; and cost-effectiveness.

##### Multidimensional phenotyping

It is suggested that the robust heritability of BD may extend to familial patterns of lithium response and several international consortia are investigating the genetics and pharmacogenomics of BD and its treatment [e.g., (26, 55)]. The R-LiNK study uses several “omic” approaches (transcriptomics, microRNA, methylomics, metabolomics, proteomics, etc.), employing a strategy that is sometimes referred to as “convergent functional omics” (56), to try to detect a molecular signature associated with biological pathways or networks underlying treatment response (57–59). However, even convergent functional omics ought not be undertaken in isolation, as a contemporary approach to integrative biology should ideally extend to a range of systems. This is especially true for a medication such as lithium, which appears to be implicated in a wide range of processes at all levels (58–60). For example, it is hypothesized that lithium inhibition of GSK-3 may result in neuroprotection and attenuation of cognitive deficits (57) as well as modification of circadian clock machinery (28), etc. Findings on neuroplasticity build a bridge to neuro-imaging research which in turn examine neuro-anatomical and biochemical abnormalities associated with a diagnosis of BD and the effects of lithium on brain structure, biochemistry, and connectivity in BD-I [e.g., (61–63)]. Likewise, hypotheses regarding circadian rhythms may be linked to actigraphic examination of sleep-wake cycles [e.g., (64, 65)]. Merging findings within and across these dimensions may lead to the identification of combinations of biomarkers or biosignatures, with greater predictive value than isolated markers (54).

Several research groups have suggested that it is worthwhile to extend the search for phenotypes beyond the established architypes (66). In R-LiNK, this includes strategies such as exploring the heterogeneity in brain lithium distribution and whether this differs in responders and non-responders (67). However, it may extend further to new paradigms such as smartphone apps and wearable technologies, which can be employed to assess digital phenotypes (a term that describes health data collected from individual monitoring, social media use, and measurement of interactions with technology e.g. the combination of self-rated PRO, EMA, and actigraphy) (68, 69). In BD, this has the potential for real-time recording of mood, activation and sleep-wake cycle, and the early detection of any changes in symptoms or health status parameters that are associated with lithium treatment (42, 46).

**Figure 1:**
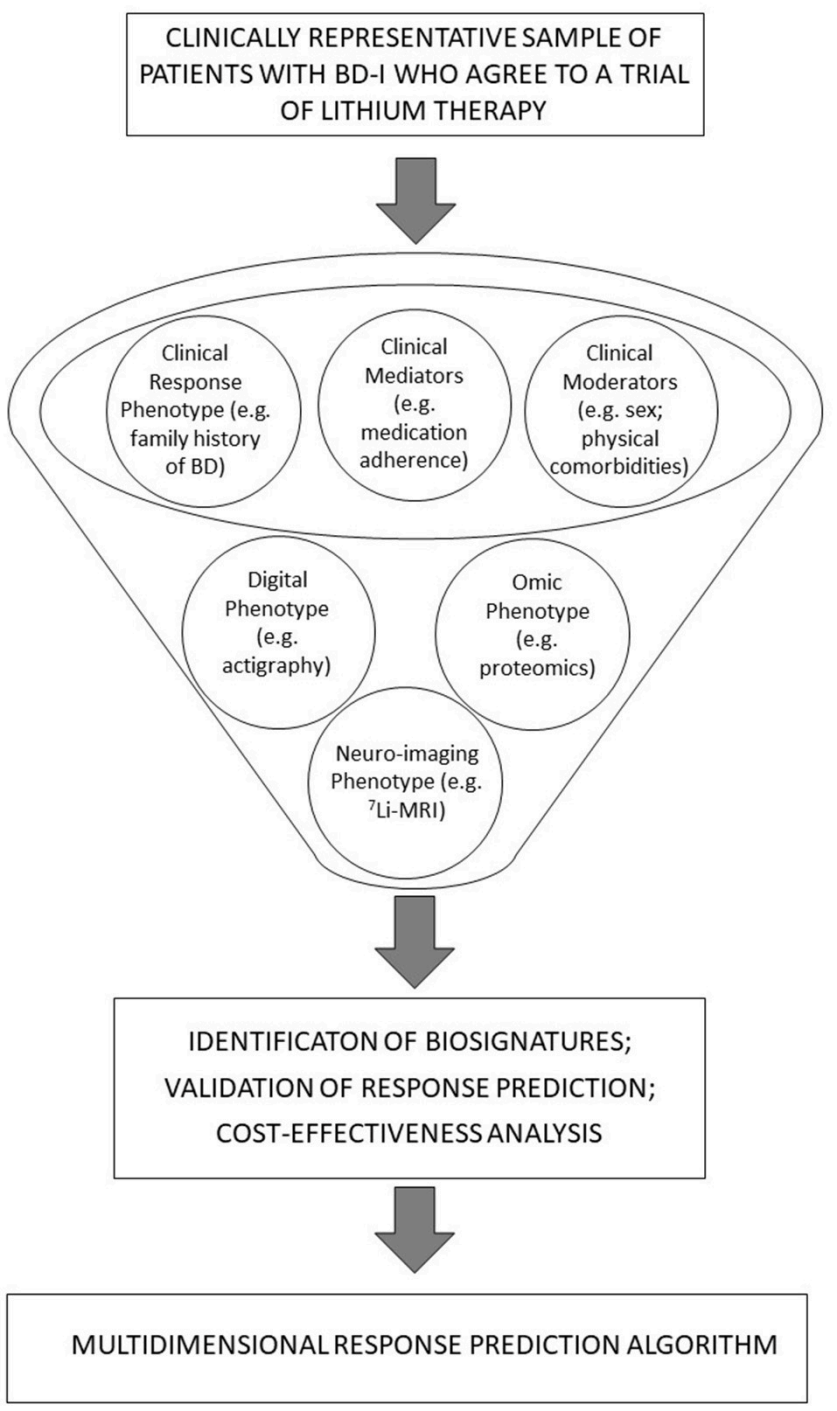
A diagrammatic representation of integrated, bottom-up and top-down approaches and their potential role in the development of precision prescribing pf lithium in BD-I.

Whilst multidimensional precision modeling for response to lithium in BD-I is in its infancy, it is noteworthy that this strategy is employed increasingly in psychiatry research. For example, some research on suicidality has examined clinical and biological markers and combined these into an algorithm for predicting suicide attempts (70). With this in mind, Figure 1 offers a diagrammatic representation of the steps involved in using this approach for precision prescribing of lithium. The figure tries to include core elements of the top-down and bottom-up approaches as shows approximately the point at which each of these elements might be considered.

##### Analytic strategies for response prediction

A biosignature can be based on a combination of biomarkers and clinical characteristics (71) and their joint contributions to the prediction of different dimensions of treatment response. As shown in Figure 1, these may be further examined from the perspective of moderators and mediators (72); e.g., moderators of treatment effects may include baseline clinical characteristics (e.g., gender, family history of BD, etc.). Likewise, early markers of lithium response may be derived from tests undertaken in the weeks or early months after treatment is initiated (e.g., brain lithium distribution after 12 weeks of lithium). Potential mediators of lithium effects may include changes in omics or neuroimaging markers that occur over a short timeframe (e.g., changes in variables measured immediately prior to and 12 weeks after commencing lithium) but could also include early changes in sleep-wake cycles as shown by actigraphy recordings or EMA symptom ratings (21, 42, 46).

Statistical analyses in precision psychiatry research are complicated by the fact that many studies rely on “high dimensional data,” i.e., many measured variables (repeated measures of multiple putative biomarkers) collected in a relatively restricted clinical sample (of a few hundred participants or sometimes less). Whilst it is feasible to construct a treatment decision algorithm from such data, considerable statistical expertise is required to address the challenges arising regarding data management, harmonization of data on biomarkers derived from different systems and handling missing data within and across domains, as well as avoiding “over-fitting” of statistical models, such as machine learning (73). The latter is increasingly being applied to psychiatry in general and BD in particular to aid diagnostic and treatment selection (74, 75).

Many researchers argue that discovery science strategies may be justifiable approaches to analyzing data in precision psychiatry as these are both hypothesis generating as well as hypothesis-testing [e.g., (56, 76, 77)]. For instance, a comprehensive stepwise statistical approach to outcome prediction (starting with discovery, prioritization and validation, followed by further examination of selected predictors) may lead to the discovery of novel biomarkers as well as replicating findings from previous research and ultimately lead to the development of a prediction tool (70). A similar strategy may be useful for R-LiNK or comparable studies, which attempt to quantify the predictive value of putative biomarkers and to determine which combinations of markers have additive or interactive effects for identifying an individuals' likelihood of treatment response.

In practice, we suggest that contemporary studies may need to combine discovery science approaches for the identification of putative early predictors of lithium response (defined as a categorical outcome) with more targeted analyses focused on the exploration of markers of different measures of response (e.g., time to achieve a response category; continuous measures of response such as number of days ill per annum for 2 years before and after lithium initiation). This strategy allows researchers to consider the level of precision of biomarkers by using predetermined approaches to analyzing any biosignatures associated with different definitions of response. For example, machine learning is now widely employed for pattern recognition, and multivariate logistic regression or mixed models might be used for analyses of categorical measures of response. Other concepts of response, such as the analysis of PRO might focus on temporal changes in quality of life or employ “mirror-image” approaches to change in symptoms or health status over time. More subtle notions of response, such as intra-individual, day-to-day symptom variability (as measured by the digital phenotype) will require different statistical models and, e.g., actigraphy data may be best explored using non-linear dynamics.

##### Cost-effectiveness

Another, so far ignored issue in precision prescribing in psychiatry, is the need to determine whether the cost of biomarker-driven treatments will be economically as well as clinically justifiable. For example, even if a multidimensional predictor tool or algorithm is developed for predicting response to lithium and shows external validity in replication studies, very expensive testing of large populations may complicate value assessments and, in some instances, may indicate that the use of the tool is unlikely to be cost-effective (78).

To better inform clinical decision-making, precision psychiatry research will need to determine which biomarkers or biosignatures can be transferred most efficaciously from bench to bedside. This translation should be assessed from several perspectives, including the positive and negative predictive values of different sets of biomarkers, additivity, or redundancy in employing multimodal biosignatures, access to and interpretability of tests, as well as patient acceptability or burden associated with testing, etc. This process represents a critical step and needs greater acknowledgment in research planning and reporting of findings in future publications. These translational steps can be considered alongside cost-effectiveness by constructing simulation models that estimate the expected lifetime costs of treatment for BD-I (of which lithium is one element) compared to the predicted outcomes in terms of Quality Adjusted Life Years.

## Discussion

Clinical syndrome subtyping has failed to inform personalization of treatments in psychiatry (10, 19, 79). This is partly because even more narrowly defined clinical presentations, such as those included in the BD-I category still represent heterogeneous endpoints of different underlying causal pathways. The latter may include clinical, demographic and environmental factors, and genetic, epigenetic and other biological processes (19, 20); but this diversity also offers a plausible reason to promote personalized approaches rather than avoid exploring them (8, 19).

The search for biomarkers of psychotropic treatment response is in its infancy (80), but there are encouraging emerging findings form individual studies and useful up-to-date syntheses of the data in several existing systematic reviews (8–12, 38). This narrative review seeks to argue that now is an ideal time to consider the development of a robust template for such studies so that the approach can be applied to research on existing treatments and then to much needed new drug developments in the future (81). Although the prediction of lithium response remains ambiguous (26), it is hoped that the identification of a combination of clinical and biological markers may inform the development of a composite prediction algorithm that could guide treatment decisions in BD-I (37, 72). The additional advantage of considering lithium is that we have some information on plasma levels associated with therapeutic response as well as toxicity, which is not the case for most other putative mood stabilizers (82). However, even starting this process involves making further progress on a consensus of how to best monitor illness activity (to assess changes between pre-to-post-lithium initiation periods) and best define response. This step is needed to enhance not only the quality of research (validation of putative predictors) but also to assist in the translation of findings from biomarker research into the clinical application of biosignatures in day-to-day practice.

Precision prescribing of lithium holds the promise of reducing the duration of a treatment trail from about 18–24 months to 3 months or less (as response might be predicted prior to commencing lithium or within the typical timeframe for titration of the dose of lithium). However, widespread dissemination into clinical practice of one or more biomarkers or biosignatures will require synergy between academia and industry and government (83). The incremental cost effectiveness of these strategies is likely to change significantly over time, as the cost of biomarker assays may reduce, but the cost of introducing new medications increases. Thus, markers that increase the prediction of treatment response are likely to become more valuable over time. Furthermore, as noted by Fernandes et al. (18), merging research findings into EBM-driven or “personalized” CPG will require the addition of new sections within the guidelines that specify how any newly developed technologies should be employed and further evaluated in clinical settings.

This review was undertaken in order to more fully appreciate the broader concepts and emerging strategies being employed in biomarker research. This was deemed important because nearly all existing reviews on this topic in BD focused on study findings rather than considering whether the research strategy or methodology was a source of confounding or variance between studies. Overall, this narrative review suggests that the opportunities for research and development of precision prescribing of lithium in BD-I must be balanced by a realistic appraisal of the complexity of analyzing high dimensional data, the robustness of any putative biosignatures identified and the potential barriers that will then arise when moving forward to clinical implementation. Currently, high quality biological research will be undermined, and translational options will be reduced without additional consideration of integrative approaches, including the clinical evaluation, analytic strategy and how biosignature findings can be introduced efficiently into real world settings.

## Ethics statement

The R-Link study is being carried out in accordance with the Declaration of Helsinki; ethical approval for each of the centres is granted by the relevant ethics committee in that country.

## Author contributions

All authors contributed substantially to the preparation of the manuscript. JS and FB undertook the literature review. JS wrote the first draft of the manuscript, BE revised the draft manuscript. FB (the chief investigator for R-LiNK) added relevant insights gained from the development of the R-LiNK project. All authors contributed to writing the submitted draft and approved the final version of the manuscript.

### Conflict of interest statement

All authors have completed the ICMJE disclosure form. The authors are members of the R-LiNK initiative which is supported by an EU H2020 grant, however, the interpretation of the literature and opinions expressed in this review are those of the authors and do not represent those of grant funding agency.
